# Could Conservation Strategies for the Hainan Gibbon Generate an Umbrella Effect for the Hainan Four-eyed Turtle?

**DOI:** 10.3390/ani16121813

**Published:** 2026-06-12

**Authors:** Fanrong Xiao, Zihang Cai, Shigang Luo, Tien Ming Lee

**Affiliations:** 1 Ministry of Education Key Laboratory for Ecology of Tropical Islands, Key Laboratory of Tropical Animal and Plant Ecology of Hainan Province, College of Life Sciences, Hainan Normal University, Haikou 571158, China; 202512071300001@hainnu.edu.cn (Z.C.);; 2 Hainan Institute of National Park, Haikou 570203, China; 3 State Key Lab of Biological Control, School of Ecology, Sun Yat-Sen University, Shenzhen 518000, China

**Keywords:** Hainan Tropical Rainforest National Park, freshwater turtle, relative population densities

## Abstract

Umbrella species can facilitate efficient biodiversity conservation, as their targeted protection effectively safeguards co-occurring species and entire ecosystems in conservation management. To verify Hainan gibbon (*Nomascus hainanus*) as an umbrella species for sympatric endangered species, this study evaluated the conservation efficacy of the Hainan four-eyed turtle (*Sacalia insulensis*) through multidimensional spatiotemporal analysis. By comparing the population data collected using cage-trapping methods from 2005 to 2025, the *S. insulensis* population in the Bawangling region of the Hainan Tropical Rainforest National Park has exhibited obvious recovery, from no initial records to 25 individuals (mean relative population density: 0.0404 individuals/cage-day). Its growth trajectory is closely linked to the continuous enhancement of conservation efforts for Hainan gibbons, confirming umbrella effects stemming from poaching suppression and habitat restoration. However, when compared with recent surveys in other park regions, significant spatial limitations were revealed that the mean relative population densities at Yinggeling (0.01 individuals/cage-day) and Diaoluoshan (0.0064 individuals/cage-day) were only 25.74% and 15.84%, respectively. Additionally, juveniles constituted only 12% of Bawangling’s population, indicating that while the umbrella strategy delivers meaningful conservation outcomes, supplementary targeted initiatives are still needed to improve efficiency in resolving the species reproductive bottlenecks (e.g., nesting habitat quality). Those findings demonstrate that umbrella species conservation can establish an ecological conservation force field; however, its effectiveness is constrained by unequal resource allocation and species niche differences. Future efforts should overcome the spatial and ecological limitations of single-species umbrella effects by using smart patrol networks, secondary umbrella species mechanisms, and dedicated management plans.

## Simple Summary

Research assessed if protecting Hainan gibbons also aids endangered Hainan four-eyed turtles. Within the gibbons’ protected area (Bawangling), turtle numbers grew from zero to 25 over 20 years, confirming a positive umbrella effect from reduced poaching bonded to habitat restoration. This study is the first to validate that Hainan gibbons exert an umbrella effect on sympatric reptiles. It verifies the practical outcomes of existing conservation work, identifies tangible ecological changes brought by current protection strategies, and provides evidence and inspiration for further conservation actions and relevant research in Bawangling of Hainan Tropical Rainforest National Park.

## 1. Introduction

Owing to the limited funding, knowledge, and time available for conservation actions, efforts often rely on science-based shortcuts to maximize biodiversity conservation efficiency [1,2]. The “umbrella species” concept, a strategy for guiding spatial conservation planning, considering the needs of a given species, has gained significant attention [3]. Its core premise is that protected areas and management measures designed to support an umbrella species (typically those with extensive or specific habitat needs) will concurrently safeguard coexisting species within the same landscape [4,5]. This approach is widely regarded as time- and cost-effective, particularly in data-poor regions or areas which require urgent conservation actions [6,7].

However, empirical research reveals that the practical effectiveness of umbrella species strategies continues to generate controversy conditions [8]. Several studies indicated that specific large or wide-ranging species can effectively serve as umbrellas. For example, Studies on fishes and birds demonstrated that umbrella species can accommodate the conservation needs of co-occurring species in habitat conservation [7,9]. On the other hand, another studies highlighted inconsistent or non-universal outcomes [5,10,11]. Despite not meeting the full requirements of all co-occurring species [12,13], the conservation benefits conferred by umbrella species are evident in analyses of these associated communities, providing a robust foundation for further research into community-level conservation needs.

The Hainan gibbon, *Nomascus hainanus*, is an endemic species, also classified as critically endangered, distributed on Hainan Island, China [14], has been designated as both a flagship and umbrella species of the Hainan Tropical Rainforest National Park [15]. As apex consumers that require vast tracts of primary forest, stringent protection measures for these species should theoretically shield forest ecosystems and numerous sympatric species [16]. Currently, only seven groups remain, a total of approximately 44 individuals [17]. To rescue this species, the Bawangling region implements rigorous interventions including: intensive anti-poaching patrols, isolation protection of core habitat zones, and long-term monitoring [18]. Consequently, it has been widely recognized that these measures have also passively or actively conferred umbrella protection to other endangered flora and fauna and their rainforest habitats in the region [16]. However, robust empirical evidence confirming the scope, intensity, and taxonomic effectiveness of this umbrella effect remains scarce, especially for species of low conservation concern.

Testudines represent the vertebrate group with widely global extinction risk after Primates [19]; however, they have long suffered from conservation neglect that is disproportionate to their threatened status. Globally, over 60% of all chelonian species now face extinction threats, far exceeding the comparable risks for mammals and birds [19,20]. This crisis is particularly challenging in China, where over 92% of native turtle species are listed as endangered or critically endangered [19], mainly leaded by extensive anthropic actions in the region [21,22].

Hainan hosts 12 native freshwater turtle species, accounting for 40% of China’s non-marine chelonian diversity [23]. Alarmingly, 83% (10 species) of these are currently classified as endangered or critically endangered by the IUCN Red List. The Hainan four-eyed turtle (*Sacalia insulensis*) epitomizes this crisis. Originally identified as *Sacalia quadriocellata*, it is nationally designated as a Class II Protected Species [24] and is classified as endangered by IUCN [25]. Molecular phylogeographic and morphological studies have revealed that the Hainan populations represent distinct species [26]. Historically widespread in island streams at altitudes of 170–939 m [27,28,29,30], this species is now severely affected by the extensive destruction of primary tropical rainforest habitats and prolonged intensive illegal harvesting [31]. Wild populations have undergone a dramatic decline and have become highly fragmented [31]. It is currently confined primarily to the less disturbed areas of the Hainan Tropical Rainforest National Park, and it ranks among the park’s 90 prioritized vertebrates (Top 9) [15].

Given the Hainan gibbon’s role as an umbrella species, the significant conservation investment focused on creating a strict sanctuary at Bawangling, and the fact that the cryptic and highly poachable *S. insulensis* coexists in rainforest habitats, this study examined whether the gibbon’s protection effectively benefits this imperiled turtle. This synthesis provides vital scientific evidence for optimizing management efficacy in the Hainan Tropical Rainforest National Park and advancing real-world applications of the umbrella species theory in multi-tiered biodiversity conservation, particularly for neglected and highly threatened non-focal species.

## 2. Materials and Methods

### 2.1. Study Area

**Figure 1:**
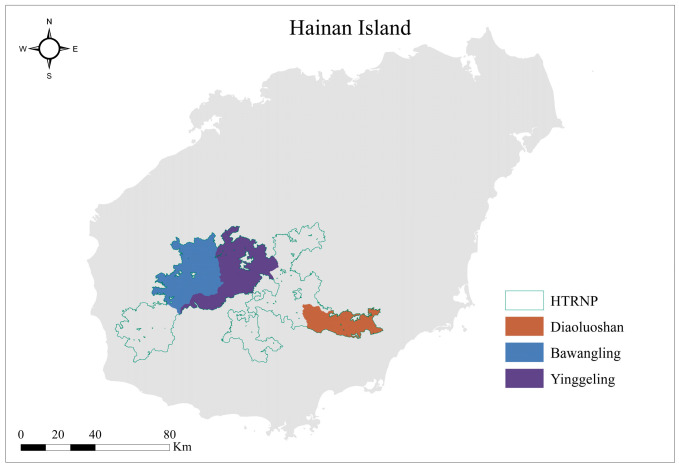
Location of Bawangling, Yinggeling, and Diaoluoshan region in the Hainan Tropical Rainforest National Park (HTRNP), Hainan Island, China.

Hainan Tropical Rainforest National Park is located in the central part of Hainan Island, with a total demarcated area of 4269 km^2^ [32]. It represents the most concentrated, most diverse, best-preserved, and largest contiguous area of continental island tropical rainforest in China. Hainan Tropical Rainforest National Park comprises seven regions. This study was conducted in the Bawangling, Yinggeling, and Diaoluoshan regions (Figure 1). The Bawangling region is situated in the southwestern part of Hainan Island. Its area within the national park is 857 km^2^. The Diaoluoshan region is located in the southeastern part of Hainan Island, specifically within the eastern section of Hainan Tropical Rainforest National Park. Its area is 447 km^2^. The Yinggeling region lies in the central-southern part of Hainan Island with a total area of 861.7 km^2^, it serves as the central nexus of Hainan Tropical Rainforest National Park.

### 2.2. Literature Review

Historical *S. insulensis* (or *S. quadriocellata*) population data in the Bawangling region were obtained from a comprehensive literature search. This encompassed peer-reviewed and gray literature retrieved from the China National Knowledge Infrastructure, Google Scholar, and Chinese university theses/dissertations published between 1995 and 2025.

### 2.3. Cage-Trapping Methodology

Between 2020 and 2025, field surveys were conducted across stream habitats at elevations of 111–700 m in three regions of the Hainan Tropical Rainforest National Park: Bawangling, Diaoluoshan, and Yinggeling. Three streams in the General Control Area were selected from each region. These streams were selected based on consultations with local rangers, focusing on streams with historical records of *S. insulensis* presence.

Specifically, sampling employed a systematic, equally spaced approach wherein nylon cages (height = 20 cm, diameter = 40 cm, mesh size = 1.2 cm) were deployed along predetermined stream transects at 30–50 m intervals. Each cage was placed for 3–5 days. The cages were baited with either dried salted fish or fresh pork liver to attract turtles.

All cages underwent daily morning inspections (between 07:00 h and 10:00 h), with systematic recordings of the cage site elevation, transect length, captured individual counts, and trapping effort (days and number of cages deployed). For every captured specimen, the straight-line carapace length was measured and the sex/age class (adult/juvenile) was documented. Native species were then released immediately at the capture points, whereas non-native species were removed from the wild ecosystem. Upon completion of the survey, all cages were retrieved to prevent inadvertent harm to the local wildlife.

The population density of the turtles was estimated as the relative population density (RPD; catch per unit fishing effort) using the following formula:

RPD = n/cage-day

where n is the number of turtles caught and cage-day is the number of cages multiplied by the number of days.

### 2.4. Statistical Analysis

All statistical procedures and graph generation were performed in SPSS (version 16.0; SPSS Inc., Chicago, IL, USA). Differences were considered statistically significant at *p* < 0.05. Due to the recent RPD data (2020–2025) of the Bawangling, Diaoluoshan, and Yinggeling regions showing a normal distribution (Kolmogorov–Smirnov test) and homogeneity of variance (Levene test), the RPD differences among Bawangling, Diaoluoshan, and Yinggeling regions were analyzed using LSD post hoc tests in One-way ANOVA.

## 3. Results

**Figure A1:**
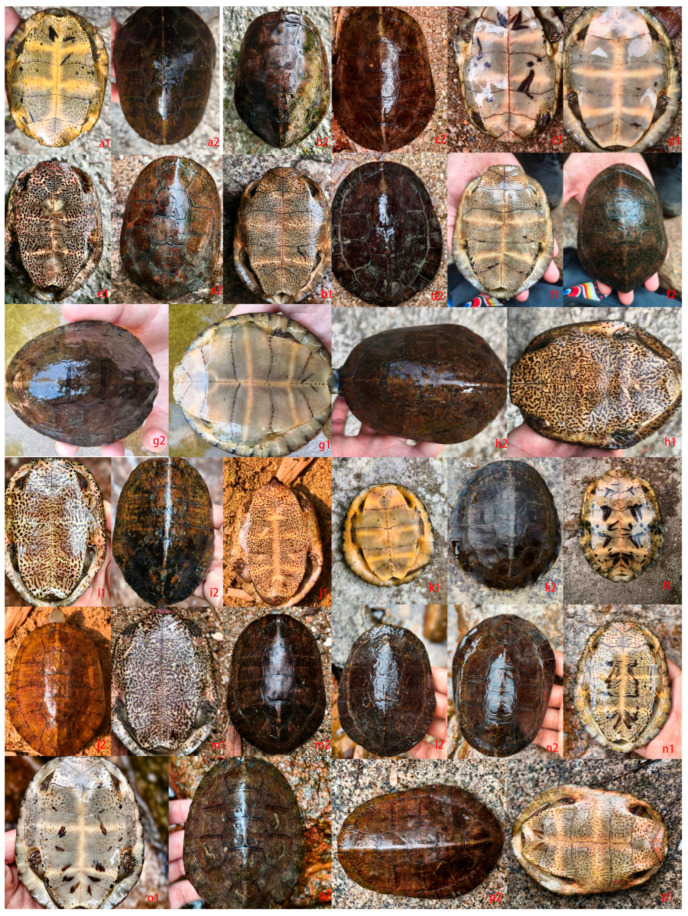
Some individuals of *Sacalia insulensis* captured in Bawangling, Hainan Tropical Rainforest National Park. The same lowercase letter (**a**–**p**) represents the same individual, with 1 and 2 representing the plastron and carapace, respectively.

A literature review display that in June and July 2005, 750 cage-days were spent in 8 km streams at an altitude of 400–1000 m in Bawangling; however, no turtles were found [27]. In 2025, from March to April, cage trapping survey comprises 7.77 km across three streams in eastern Bawangling National Park, documenting 25 individuals (Table 1), being 11 adult females, 11 adult males, and three juveniles (Table A1; Figure A1). This resulted in a mean relative population density of 0.0404 ind./cage-day (SD = 0.0108). Compared to Gong’s null records from eastern Bawangling [27], the current findings demonstrate slightly population recovery. Although the two years surveyed have a little inconsistency, both have overlap of most elevations. Especially the elevation where turtles were caught (391–580 m), the two years basically both include these. In addition, the two years’ surveys were all in the same area of Bawangling (Dongyi Area).

**Figure 2:**
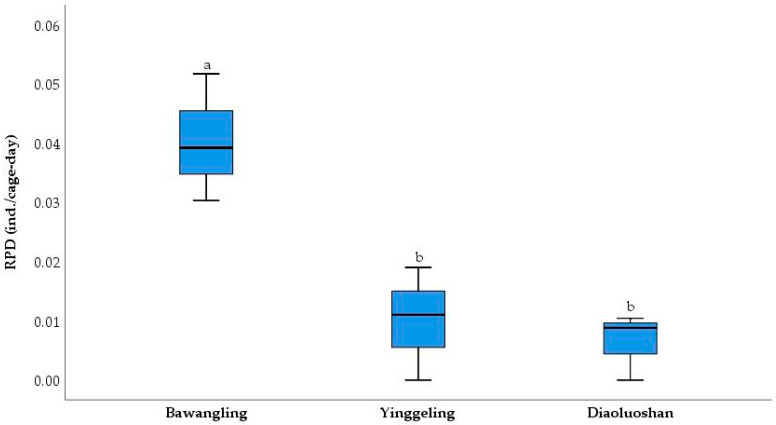
The relative population density (RPD) of *Sacalia insulensis* differs between Bawangling, Yinggeling, and Diaoluoshan based on data from 2020 to 2025. The lowercase letters on the box plot denote differences based on LSD post hoc tests in One-way ANOVA (*p* < 0.05). RPD: catch per unit fishing effort.

Regional analysis further revealed that Bawangling’s density exceeded Yinggeling four-fold and surpasses Diaoluoshan six-fold, recorded in 2020–2025 in these regions of national park monitoring data (Table A2 and Table A3). Moreover, there were significant differences in the relative population density among the three regions (F = 13.196, *p* < 0.01; Figure 2).

## 4. Discussion

During a 20-year period (2005–2025), *S. insulensis* population in Hainan’s Bawangling exhibited obvious demographic shifts. The 2025 spring survey recorded 25 individuals at a mean relative density of 0.0404 ind./cage-day, a definitive recovery from Gong’s null records in the same region of Bawangling. This positive population trajectory demonstrates close temporal concordance with intensified Hainan gibbon conservation measures, commencing with the 2003 reserve expansion (66.26–299.80 km^2^), which enabled substantial funding allocation, enhanced patrol coverage, and strengthened anti-poaching enforcement, among other initiatives [18,33]. Subsequent incorporation into the national park system in 2021 will further amplify protective measures [32]. These dynamics provide temporal evidence of an umbrella effect, whereby non-target species within the habitats of flagship-protected species gain indirect benefits through poaching deterrence and habitat recovery [18]. The longitudinal results of Bawangling substantiate the idea that flagship species conservation generates protective cascades across ecological communities.

*S. insulensis* is a typical stream-dependent species, highly sensitive to forest integrity, riparian vegetation structure, water cleanliness and human disturbance intensity [27]. Habitat fragmentation, water pollution and illegal hunting are its major threat factors. In the Bawangling area, a series of measures implemented for the population recovery of Hainan gibbons, including full-scale forest conservation, regular patrol and law enforcement [18], may have effectively mitigated the key survival threats to *S. insulensis* and preserved high-quality stream microhabitats suitable for its foraging. This finding verifies that targeted conservation of rare primates can realize the coordinated protection of stream-dwelling species through overall habitat management.

However, population comparisons across spatial scales (Bawangling vs. Yinggeling and Diaoluoshan) revealed limitations in the umbrella effect. Despite the shared Hainan Tropical Rainforest National Park governance, Bawangling’s *S. insulensis* relative density was four times higher than that of Yinggeling and six times higher than that of Diaoluoshan. This disparity cannot be fully explained by natural habitat heterogeneity, as this species was historically widespread across islands [28]. The root cause lies in spatial disparities in conservation resource allocation (financial and human resources), which creates differential protective efficacy [18]. As the last habitat of Hainan gibbons, Bawangling receives substantially greater patrol funding and research investment than other regions, with a higher infrared camera coverage density [18]. This flagship species siphoning effect results in significantly suppressed anthropogenic harvesting threats within Bawangling, whereas other regions face persistent capture pressure due to weak patrol enforcement [31].

The Hainan gibbon relies exclusively on primary forest canopies above 800 m elevation [34,35], whereas *S. insulensis* mainly inhabits stream corridors in 170–640 m lowland zones coinciding with high-intensity human activity at reserve edges [28]. Strict gibbon conservation in Bawangling incidentally shelters these lowland freshwater habitats; however, anthropogenic disturbances persist in the low-elevation sectors of other regions [28]. This explains conservation failure beyond Bawangling: when flagship species fail to drive protection investments in marginal habitats, ecologically specialized threatened species remain exposed to risks.

Hierarchical protection gaps exist even within the Bawangling region. Juvenile *S. insulensis* comprised only 12% (3/25) of the Bawangling population, which is below the 30% sustainability threshold for freshwater turtles [36]. In addition, zero juveniles were detected in Diaoluoshan. This stems from species-specific vulnerabilities such as nesting beaches facing flood erosion and juveniles suffering from high predation [31]. Gibbon conservation facilitates forest recovery; however, it neglects critical microhabitat management (e.g., stream bank stabilization and nesting site maintenance). Comparatively, nest protection for wood turtles (*Glyptemys insculpta*) reduced nest destruction by 47% and increased juvenile survival by 28% [37], demonstrating that umbrella strategies alone cannot address life history bottlenecks.

Therefore, for national parks beyond Bawangling, immediate priorities should include: recognizing the urgency of establishing a flagship species coalition by designating freshwater turtles as secondary flagship species in addition to developing turtle-friendly community agreements, such as yellow-spotted river turtles (*Podocnemis unifilis*) [38]; restructuring conservation resources by integrating low-elevation streams into patrol units based on poaching/human disturbance hotspot models, while simultaneously deploying intelligent anti-poaching grids equipped with vibration sensors and drone surveillance; transcending umbrella dependence by establishing multi-species threat assessment matrices; formulating species-specific management plans for freshwater turtles under the IUCN Species Survival Commission framework; and embedding population recovery targets into Hainan National Park’s performance evaluation system.

## 5. Conclusions

In conclusion, *S. insulensis* populations in Bawangling have exhibited a slighter recovery, and their population dynamics are temporally correlated with gibbon conservation efforts. This indicates that gibbon protection curbs poaching and rehabilitates habitats via the umbrella species effect. Nevertheless, such positive outcomes are limited to the Bawangling area. To address the constraints of flagship species conservation, such as habitat niche mismatches and unmet species-specific requirements, we recommend implementing targeted microhabitat management and advancing institutional reform across Hainan’s national park system. These on-the-ground interventions will precisely cater to the ecological demands of local species, expand the coverage of conservation benefits beyond localized sites, and ultimately strengthen the overall effectiveness of regional biodiversity protection.

## Figures and Tables

**Table 1:** The number of turtles and mean relative population density (RPD) of *Sacalia insulensis* across Bawangling, Diaoluoshan, and Yinggeling in the Hainan Tropical Rainforest National Park.

Regions	Date	Method	Altitude Range (m)	Transect Length (Km)	Number of Turtles	Cage-days	RPD (ind./cage-day) Mean ± SD	Source
Bawangling	March–May, 2025	Cage-trapping	280–580	7.77	25	549	0.0404 ± 0.0108	This study
Bawangling	June, July, 2005	Cage-trapping	400–1000	8	0	750	0	[27]
Yinggeling	May–July, 2020	Cage-trapping	283–500	4.8	2	246	0.01 ± 0.0095	This study
Diaoluoshan	September 2023; April, July, 2024	Cage-trapping	300–700	4	2	305	0.0064 ± 0.0046	This study

## Data Availability

The data presented in this study are provided in Appendix A and Appendix B.

## Appendix A

**Table A1:** Age, sex, morphological measurements and capture altitude of *Sacalia insulensis* in Bawangling of Hainan Tropic National Park.

Transect	Turtle ID	Age	Sex	Carapace Length (mm)	Carapace Width (mm)	Capture Altitude (m)
Nanqi river	SY001	Juvenile	-	73.64	64.64	496
	SY002	Adult	Male	109.84	87.45	488
	SY003	Adult	Female	101.19	74.36	580
Rongshugou	SY004	Adult	Male	118.72	82.66	580
	SY005	Adult	Male	128.63	95.17	540
	SY006	Adult	Female	126.21	87.52	540
	SY007	Adult	Male	112.04	74.02	526
Wuli River	SY008	Adult	Male	122.57	88.91	562
	SY009	Adult	Male	120.52	85.81	475
	SY010	Adult	Female	111.27	83.48	473
	SY011	Adult	Female	121.50	87.71	470
	SY012	Adult	Female	91.54	74.41	470
	SY013	Adult	Male	124.96	91.81	422
	SY014	Adult	Male	126.00	90.07	403
	SY015	Adult	Female	124.92	92.88	403
	SY017	Adult	Female	124.13	88.15	351
	SY018	Adult	Male	123.77	91.62	343
	SY019	Adult	Female	132.06	91.81	343
	SY020	Adult	Male	135.54	92.99	281
	SY021	Adult	Male	123.00	86.31	281
	SY022	Adult	Female	132.54	92.14	375
	SY023	Juvenile	-	75.50	66.29	374
	SY024	Juvenile	-	85.57	74.12	374
	SY025	Adult	Female	130.71	98.86	396
	SY026	Adult	Female	129.92	93.54	391

**Table A2:** Characteristics of transects and related cage trapping data in Bawangling of Hainan Tropic National Park (March–May, 2025).

Transect	Start Elevation (m)	End Elevation (m)	Transect Length (km)	Number of Turtle	Cage-days	RPD (ind./cage-day)
Nanqi River	438	631	2.22	3	99	0.0303
Rongshugou	536	670	1.96	4	102	0.0392
Wuli River	280	581	3.59	18	348	0.0517
Total			7.77	25	549	0.1212
Mean						0.0404

**Table A3:** Characteristics of transects and cage trapping data in Yinggeling and Diaoluoshan of Hainan Tropic National Park.

Region	Date	Transect	Start Elevation (m)	End Elevation (m)	Transect Length (km)	Number of Turtle	Cage-days	RPD (ind./cage-day)
Yinggeling	May, 2020	Monangou	283	344	2.1	1 male adult	90	0.011
	May, 2020	Yashigou	347	423	1.8	0	104	0
	July, 2020	Mohao	300	353	0.9	1 Juvenile	52	0.019
		Total			4.8	2	246	0.03
		Mean						0.01
Diaoluoshan	April, 2024	Datianling	290	535	1.5	1 male adult	96	0.0104
	July, 2024	Hengling	514	759	1.9	1 female adult	114	0.0088
	September, 2023	Nanxi river	650	687	0.6	0	105	0
		Total			4	2	305	0.0192
		Mean						0.0064
